# The Role of Physical Activity as Conservative Treatment for Hip and Knee Osteoarthritis in Older People: A Systematic Review and Meta-Analysis

**DOI:** 10.3390/jcm9041167

**Published:** 2020-04-18

**Authors:** Biagio Zampogna, Rocco Papalia, Giuseppe Francesco Papalia, Stefano Campi, Sebastiano Vasta, Ferruccio Vorini, Chiara Fossati, Guglielmo Torre, Vincenzo Denaro

**Affiliations:** 1 Department of Orthopaedic and Trauma Surgery, Campus Bio-Medico University of Rome, 00128 Rome, Italy; r.papalia@unicampus.it (R.P.); g.papalia@unicampus.it (G.F.P.); s.campi@unicampus.it (S.C.); s.vasta@unicampus.it (S.V.); f.vorini@unicampus.it (F.V.); g.torre@unicampus.it (G.T.); denaro@unicampus.it (V.D.); 2 Department of Movement, Human and Health Sciences, University of Rome “Foro Italico”, 00100 Rome, Italy; chiara.fossati@uniroma4.it

**Keywords:** physical activity, active exercise, sport, land-based, aquatic, knee or hip osteoarthritis, older people, systematic review, meta-analysis

## Abstract

The aim of this systematic review and meta-analysis is to determine the role of physical activity as a conservative treatment for older people with knee or hip osteoarthritis. The effect on pain, physical function, stiffness, quality of life, and dynamic balance of Aquatic Exercise, Land-based Exercise, and Sports were compared in a specific population composed of osteoarthritic patients aged 65 or over. A systematic search using Pubmed-Medline, Google Scholar, and the Cochrane Library was carried out to select randomized clinical trials, observational studies, or case series that evaluated outcome measures after physical activity. Twenty randomized controlled trials (RCTs) and two case series were included in this review. Four trials were at low risk of bias (A), 12 at unclear risk of bias (B), and four at high risk of bias (C). Compared to controls, Aquatic Exercise, Land-based Exercise, Tai Chi, and Yoga showed a small to high effect for improving pain, physical function, quality of life, and stiffness. Active exercise and sport are effective to improve pain and physical function in elderly people with osteoarthritis. Nevertheless, further studies are required to validate the use of land-based exercise, aquatic exercise, or sport to treat the symptoms of older adults that suffer from knee and hip osteoarthritis.

## 1. Introduction

Osteoarthritis (OA) is a chronic progressive disease that represents a considerable cause of impairment in elderly people [1]. It is characterized by pain, reduction of physical function with decreased range of motion (ROM), joint rigidity and swelling, muscle weakness, and joint instability [2,3]. All these conditions lead to impaired quality of life with worsening to achieve daily activities and disability, especially in older adults [4]. Knee and hip are commonly affected by OA [5] because they represent the joints most involved in heavy weight-bearing and increased activity [6,7]. The prevalence of OA is higher in women and elderly people [8]. OA requires remarkable healthcare resources and involves considerable social costs for treatment, due to its progressive and chronic condition, and those demands are bound to increase with an aging population [9]. Conservative treatment for OA consists of pharmacologic therapy (Non-Steroidal Anti-Inflammatory Drugs, cyclooxygenase inhibitors, oral or transdermal opioid, acetaminophen), injective therapy (corticosteroids, Hyaluronic acid, Platelet-Rich Plasma or Adipose-Derived Stem Cell), supportive therapy (glucosamine or chondroitin), physical therapy (Electrical Nerve Stimulation, Pulsed Electromagnetic Field, Laser Therapy, Therapeutic Ultrasound), braces, orthoses, active exercise (aquatic or land-based), physical and sport activity [10,11,12]. We focused our research on active exercise and sports that have been determined to be effective in pain relief, maintenance of joint integrity, and muscle strength, improvement in physical function, and lessening deformity and instability [13]. Active exercise has been proved to increase physical function and reduce knee and hip pain and disability, improving general health status and quality of life [14,15]. The role of exercise or sportive activities is to avoid or delay the necessity to recur to the knee or hip joint replacement, which must be reserved just for the final stage of OA [16], which is characterized by severe pain and deformity. Land-based exercise programs such as aerobic, strengthening, and resistance training are effective therapies for knee and hip OA [17,18]. Furthermore, the increase of lower limb muscular strength and the improvement of balance and coordination of movements are effective in achieve compensatory functional stability in older people with advanced OA in order to reduce the risk of falling [19,20]. Aquatic exercise profits by the weight-relieving properties of water to obtain pain relief, to allow easier joint movement improving physical function, to reduce muscle stiffness and to cause muscle relaxation in patients with OA [21,22]. In contact sports, there is a higher incidence of significant joint injuries or progression of osteoarthritis [23]; however, low-impact sports are suggested as a physical treatment for osteoarthritis because they prevent from maximum stress and enhance muscle strength and joint stability [24]. Therefore, sports are effective both in the prevention and in the treatment of OA, but they have to be modulated on the individual patient’s physical abilities. The evidence on comparative effectiveness on pain and physical function of different types of active exercise or sport interventions or older adults with knee or hip OA is still poor. In fact, an adequate activity to conservatively treat those patients obtaining clinical benefits has not yet been identified. Therefore, the aim of this systematic review and meta-analysis is to determine the efficacy of physical activity as a conservative treatment for elderly people with knee or hip OA. The primary endpoint is to assess the effect on pain, physical function, stiffness, quality of life, and dynamic balance outcomes of different active exercise and sports. The secondary endpoint is to establish the specific benefits on the selected outcomes of the single intervention, to try to evaluate if there is an exercise or sport that leads to better enhancement in physical capacity and quality of life of older osteoarthritic adults.

## 2. Materials and Methods

A systematic review and meta-analysis were carried out using the Preferred Reporting Items for Systematic Reviews and Meta-analysis (PRISMA) guidelines [25]. The review was planned and conducted following the PRISMA checklist (Supplementary Materials Table S1 ). In this review, we included randomized clinical trial, observational studies, or case series, which evaluated the role of sport or exercises as a conservative treatment for patients aged 65 or over with all degrees of the knee and hip OA.

### 2.1. Criteria for Considering Studies for This Review

According to the WHO definition of the elderly, studies with patients with a mean age of 65 or over both in the experimental group(s) and the control group, if present, were included in the review. Studies that compared effects on pain, physical function, and physical performance of the aquatic exercise, land-based exercise, or sports with a control group were included in the review. In the randomized clinical trial, the patients in the control group had to receive usual care or no intervention. We excluded studies that investigated physical activity in the prevision of hip or knee surgery or after hip or knee replacements.

### 2.2. Search Methods for Identification of Studies

A systematic literature search was performed using the following databases: Pubmed-Medline, Google Scholar, and the Cochrane Library. For Pubmed we used the following search strategy: (“exercise”[MeSH Terms] or “exercise”[All Fields] or (“physical”[All Fields] and “activity”[All Fields]) or “physical activity”[All Fields]) and (“knee osteoarthritis”[MeSH Terms] or “ knee osteoarthritis”[All Fields]); (“exercise”[MeSH Terms] OR “exercise”[All Fields] or (“physical”[All Fields] AND “activity”[All Fields]) or “physical activity”[All Fields]) AND (“hip osteoarthritis”[MeSH Terms] or “hip osteoarthritis”[All Fields]). The reference list of the identified articles was screened manually for further publications. After duplicates removed, the abstracts of all studies eligible were independently examined by two review authors (G.P. and B.Z.). Any uncertainties or disagreements (17 in total) were discussed with the third reviewer (R.P.) to reach a consensus. Two reviewers (G.P. and B.Z.) screened the full articles in order to determine those to include in the review and quantitative analysis.

### 2.3. Data Collection, Analysis, and Outcomes

Data extraction was independently produced by two reviewers (G.P. and B.Z.). We extracted the following study characteristics: authors, year of publication, type of study, level of evidence, numbers of participants in the intervention or control group integrated with age, gender and Body Mass Index (BMI), joint(s) involved, intervention in the study and in the control group, primary and secondary outcome measures, follow-up, and results. Any uncertainties or disagreements (four in total) were discussed with the third reviewer (R.P.) to reach a consensus. Outcomes included the severity of pain, which was measured on a visual analog scale (VAS), on the pain scale of the Western Ontario and McMaster Universities Arthritis Index (WOMAC) or the Knee injury and Osteoarthritis Outcome Score (KOOS) pain scale. Physical function was calculated by the WOMAC physical function scale, the KOOS ADL (function in daily living) scale, the 6-min walking test (6-MWT), the sit to stand test and range of motion (ROM) of the considered joint. Stiffness was checked with the WOMAC stiffness scale. The quality of life of the patients was measured through Short Form-36 (SF-36) or Short Form-12 (SF-12), and KOOS Quality of Life (QOL). Finally, the dynamic balance was assessed using the time up and go test (TUG).

### 2.4. Risk of Bias Assessment

Two review authors (G.P. and B.Z.) independently assessed the risk of bias of the randomized controlled trial (RCT) using the Cochrane Risk of Bias Tool [26]. We checked the following criteria: sequence generation, allocation concealment, blinding, incomplete data addressed, free of selective reporting, and free of other bias. Each domain was classified as presenting high risk of bias, low risk of bias, or unclear risk of bias. Then the trials were allocated to one of the following groups: low risk of bias if five or six criteria were judged adequate, unclear risk of bias if three to four criteria were judged as adequate or high risk of bias if less than three criteria were judged adequate. Two reviewers (G.P. and B.Z.) used the Methodological Index for Non-Randomized Studies (MINORS) score [27] to estimate the methodological quality of non-randomized studies. It consists of 8 items for non-comparative studies and 12 items for comparative studies. The score for each item ranges from 0 to 2, for a total maximum of 24 points.

### 2.5. Statistical Analysis

The quantitative data analysis was performed using the Review Manager (RevMan) software (Version 5.3, Cochrane Collaboration 2014, Copenhagen, Denmark). The data were pooled if at least two studies presented similar and comparable outcomes. A meta-analysis was performed to determine the effect of the different types of physical activity on pain, physical function, quality of life, stiffness, and dynamic balance. All continuous data were reported as mean difference (MD) with 95% confidence intervals when all the trials used the same score; otherwise, the standardized mean difference (SMD) with 95% confidence intervals was used when the analyzed scores were similar but not identical. Negative values of the mean difference or standardized mean difference proved the advantage of the experimental group. Heterogeneity was determined using the I^2^ test. A fixed-effect model was conducted if the I^2^ test demonstrated low heterogeneity (I^2^ < 55%); for I^2^ greater than or equal to 55%, a random-effect model was performed.

### 2.6. Quality Assessment

To assess the quality of the evidence of the outcomes presented in the reported trials, the GRADE (Grading of Recommendations Assessment, Development, and Evaluation) was performed [28]. It consists of the following five items: risk of bias, inconsistency, indirectness, imprecision, and other considerations. Each domain was defined as not serious, serious, or very serious. The resulting quality assessment of the evidence was classified as high, moderate, low, or very low.

## 3. Results

### 3.1. Results of the Search

**Figure 1:**
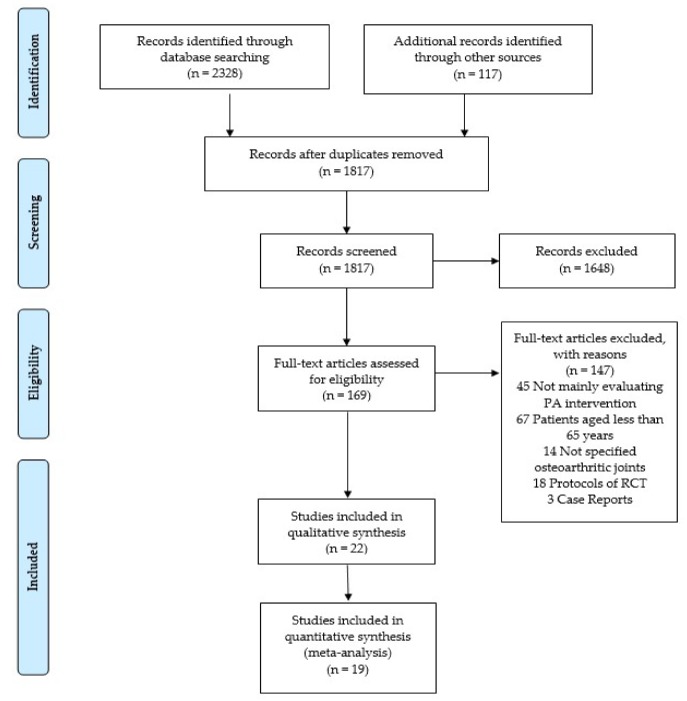
Flowchart of the article selection process.

The literature search identified 2445 articles. Of these, 1817 were screened on title and abstract after the removal of duplicates. One hundred sixty-nine articles were read in full text, and 147 of those were excluded for the following reasons: not mainly evaluating physical activity intervention (*n* = 45), patients aged less than 65 years (*n* = 67), not specified joints that suffered from OA (*n* = 14), protocols of RCT (*n* = 18), and case reports (*n* = 3). Thus, 22 articles that met the inclusion criteria were included in this review. Finally, 19 articles were included in the meta-analysis (Figure 1).

### 3.2. Included and Excluded Studies

The studies were 20 RCTs and two case series. One case series involved Baduanjin, while the other one evaluated exercise [29]. Of the RCTs, six studies checked sports activity *n* = 4 Tai Chi [30,31,32,33], *n* = 2 yoga [34,35]. The remaining RCTs were focused on water-based and land-based exercises: there were three studies with three arms (hydrotherapy, land-based exercise and control group) [36,37,38], four studies that compared only hydrotherapy with controls [39,40,41,42], and seven studies that only evaluated land-based exercises [43,44,45,46,47,48,49].

### 3.3. Demographic Data

The overall number of participants in all the studies was 1504, allocated to either intervention or control groups. The mean age of the participants ranged from 65 to 78.9 years. All studies showed a higher female percentage (ranging from 50 to 100%) of included patients. Only one study [35] did not describe gender distribution in the groups. BMI ranged between 23.7 and 33.6. All demographic data are compiled in Table 1.

### 3.4. Clinical Outcome Data

Seventeen studies included patients with knee OA alone, two studies included patients with hip OA alone, and the remaining three studies included patients with both knee and hip OA. The outcome measures evaluated in the included articles were the Western Ontario and McMaster Universities Arthritis Index (WOMAC) in 17 articles, the 6-min walk test (6-MWT) in seven articles, Knee Injury and Osteoarthritis Outcome Score (KOOS) in three articles, Visual Analog Scale (VAS) in seven articles, Short Form-36 (SF-36) in four articles, Short Form-12 (SF-12) in four articles, sit to stand test and timed up and go test in five articles (Table 2).

### 3.5. Methodological Evaluation

Using the Cochrane risk of bias tool for RCTs, sequence generation was considered adequate in 16 articles (80%), allocation concealment was graded as adequate in 15 studies (75%), blinding was inadequate in 15 trials (75%), outcome data addressed were regarded adequate in 16 articles (80%), reporting of selective outcome was judged as adequate in 14 (70%) trials, and the likelihood of other sources of bias was adequate in 10 (50%) of the studies. In conclusion, four trials were at low risk of bias (A), 12 included studies were at unclear risk of bias (B), and four studies were at high risk of bias (C) (Table 3). The MINORS score was calculated for two case series [29.50] included in the review. Only eight items were evaluated because the two studies were non-comparative (Table 4).

### 3.6. Studies Included

#### 3.6.1. Double Study Group

Five studies with two study groups were identified. Wang et al. [36] recruited 78 patients that were divided into the aquatic group, the land group, and the control group. The study demonstrated that patients in both exercise groups showed pain reduction over time. More specifically, the aquatic and the land groups presented significantly less pain than the control group at week 12 (both *p* < 0.001) and at week 6 (*p* < 0.001 for aquatic and *p* = 0.002 for land). Comparing the aquatic group with the land group, they did not show any significant difference in pain reduction at weeks 12 and 6. Foley et al. [37] evaluated 105 participants with hip or knee OA, that received water-based exercise sessions, gym-based exercise sessions, or were allocated to the control group. At follow up, walking speed and distance increased in the hydrotherapy and gym groups (both *p* < 0.001), but not in the control group. However, they did not find a significant difference between the two intervention groups for increases in physical function. Furthermore, the WOMAC pain significantly declined in the hydrotherapy group, but they did not demonstrate significant changes from baseline or between groups for WOMAC function or stiffness. Lund et al. [38] compared the efficiency of aquatic exercise and a land-based exercise program with control in 79 patients with knee OA. Only in the land-based exercise group, a decrease of pain was detected (*p* = 0.039). There were no significant differences between groups for KOOS. Fransen et al. [30] assigned 152 older patients with chronic hip or knee OA to hydrotherapy group, Tai Chi group, or a waiting list (control group). It has been shown improvements of 6.5 and 10.5 for pain and physical function scores with hydrotherapy and improvements of 5.2 and 9.7 with Tai Chi, compared with controls. Only the hydrotherapy group showed significant improvements in pain scores, SF-12, and the measures of physical performance. Cheung et al. [51] evaluated the effects of yoga and aerobic/strengthening exercises on knee OA, compared with the education control group. Patients in the yoga group presented improvements in WOMAC TOTAL (*p* = 0.001) and VAS scores (*p* = 0.03) compared to patients in exercises group.

#### 3.6.2. Land-Based Exercise

In a study by Bearne et al. [43], 48 people with hip OA were divided into the rehabilitation group or the control group. At the term of the program, the WOMAC total score improved with a moderate effect size. But there were no differences between the two groups in any outcome measure. Bezalel et al. [44] randomly assigned 50 patients with knee OA to an exercise group or a short-wave diathermy control group. At follow-up, participants in the study group showed significant improvement in the get-up-and-go test and the WOMAC total, pain, and disability scores compared to the controls (*p* < 0.01). Huang et al. [45] enrolled 250 patients with knee OA. The test group underwent quadriceps isometric contraction exercise, while in the control group local physiotherapy and oral NSAIDs were used. At three months, WOMAC and VAS scores showed significant progress in the exercise group compared to the controls (both *p* < 0.05). In a study by Doi et al. [46], 121 patients with knee OA were allocated to an exercise group and an NSAID group. The participants in both groups presented improvements in the totality of the scores (WOMAC, SF-36, and VAS at *p* < 0.001 in the exercises group; WOMAC and VAS at *p* < 0.001 and SF-36 at *p* < 0.03 in the control group), although these increases were not statistically significant between the two groups. Therefore, they showed the “noninferiority” of exercises compared with NSAIDs as a therapy for knee OA. Marconcin et al. [47] allocated 67 patients aged 60 years or older with knee OA to a self-management and exercise intervention or an educational intervention. In the self-management and exercise group, significant improvements in all KOOS dimensions (larger than 10 points) and in the 6 MWT (*p* = 0.035) were found. In a study by Hurley et al. [48], 418 practices were randomly assigned to three groups to receive usual primary care, usual primary care plus individual rehabilitation or usual primary care plus rehabilitation in groups. Six months after the end of the schedule, the WOMAC-function scores for the individual rehabilitation and group rehabilitation classes were significantly better compared with usual care (*p* = 0.01). Although the improvements were similar for participants that made individual or group rehabilitation. Takacs et al. [49] checked 40 participants that underwent exercises targeting dynamic balance and strength or no intervention. They showed significant improvement in WOMAC physical function in the exercise group (within-group *p* = 0.002; between-group *p* = 0.016). Moreover, self-reported knee pain and fear of movement results were better in the exercise group (*p* = 0.005 and *p* = 0.01, respectively) compared to the control group. In a study by Bove et al. [29], seven patients underwent a novel task-specific training approach to exercise therapy for chronic knee pain, composed of sit to stand, floor transfer, and ascending and descending stairs training. After the treatment, the participants demonstrated important improvements in both patient-rated outcomes (for example, KOOS) and performance-based outcomes.

#### 3.6.3. Aquatic Exercise

In a study by Hale et al. [39], 39 persons with hip or knee OA and at risk for falling underwent a water-based program (intervention group) or a time-matched computer training program (control group). After the 12-week intervention, they proved that water-based exercise did not decrease falls risk compared with a computer skills training class, with no significant disparities between the two groups for the primary outcome (PPA score) or any of the secondary outcomes measured. Casilda-Lopez et al. [40] divided 34 obese women with knee OA into a dance-based aquatic exercise program (experimental group) and a global aquatic exercise program (control group). Postintervention, they found significant differences between groups in the WOMAC pain and aggregate (*p* = 0.002 and *p* = 0.048, respectively) in favor of the aquatic dance group. Taglietti et al. [41] allocated 60 patients with knee OA to an aquatic exercise group, and an educational program group. After the treatment, they presented a significant decrease of WOMAC pain for the aquatic exercise group compared to the educational program group (*p* = 0.021). Furthermore, the WOMAC function decreased significantly in the aquatic exercise group compared to baseline (*p* = 0.020). Moreover, improvements in quality of life were detected in the aquatic exercise group (*p* < 0.001) at the follow-up. In a study by Arnold et al. [42], 79 adults, 65 years of age or older with hip OA were randomly divided into three groups: aquatic exercise and education, aquatic exercise only, and control. It has been described, a significant increase in fall risk factors (*p* = 0.038) for the patients the aquatics and education group, which increased in falls efficacy compared with controls. Moreover, they demonstrated a significant improvement of physical performance, Timed Up and Go Test, and 6-min walk in the aquatics and education group compared with both aquatics and control groups.

#### 3.6.4. Sport

In a study by Brismée et al. [31], 41 adults with knee OA attended a Tai Chi exercise program or an attention activities program. At follow-up, the Tai Chi group presented less overall pain and better WOMAC physical function than the control group (*p* = 0.0089 and 0.0157, respectively). Moreover, the Tai Chi group had improvements in WOMAC overall, pain subscale and physical function subscale. In a study by Lee et al. [32], 44 patients with knee OA were randomized to a Tai Chi training program or a waiting list control group. The training group reported significant increments in the total SF-36 (*p* = 0.010) and 6-m walking test (*p* = 0.005). Finally, the WOMAC scores in the training group were markedly improved, although the differences were not statistically significant. Tsai et al. [33] studied the role of Tai Chi to decrease pain and stiffness in elders with knee OA and cognitive impairment, compared with controls. They demonstrated that both groups increased their WOMAC pain score (*p* < 0.001 for Tai Chi group vs. *p* = 0.042 for control group); on the other hand, the WOMAC Physical Function and Stiffness scores improved only in the Tai Chi group (*p* = 0.001 vs. *p* = 0.515 and *p* <0.001 vs. *p* = 0.324, respectively). For all these scores, the discrepancies between the two groups improved significantly over time. Cheung et al. [34] randomly assigned 36 older women with knee OA to a yoga program or wait-list control. In their study, the differences between treatment and control groups were significant for WOMAC pain (*p* = 0.01) and stiffness scores (*p* = 0.02). An et al. [50] evaluated 22 patients (29 knees) with knee OA after one-year Baduanjin exercise. Compared with their baseline levels before exercise, patients showed significant improvements in WOMAC pain (*p* = 0.000), stiffness (*p* = 0.000) and physical function subscales (*p* = 0.003), SF-36 (*p* = 0.005), and 6-MWT (*p* = 0.036).

### 3.7. Effect of Intervention

#### 3.7.1. Pain

**Figure 2:**
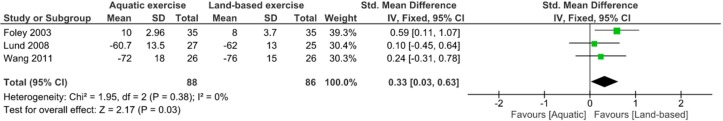
Pain: Aquatic exercise versus Land-based exercise.

**Figure 3:**
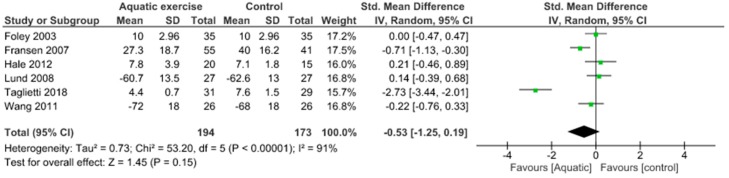
Pain: Aquatic exercise versus Control.

**Figure 4:**
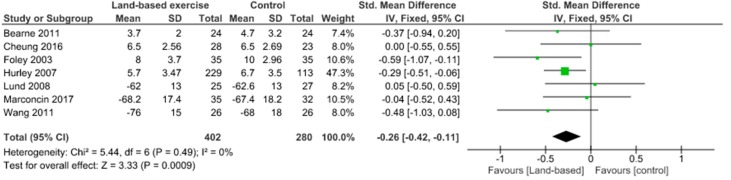
Pain: Land-based exercise versus Control.

**Figure 5:**
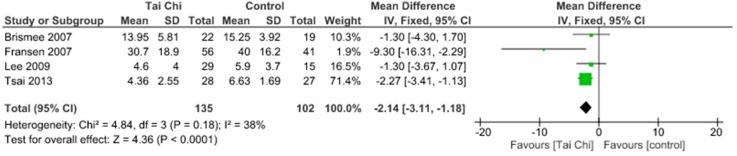
Pain: Tai Chi versus Control.

**Figure 6:**
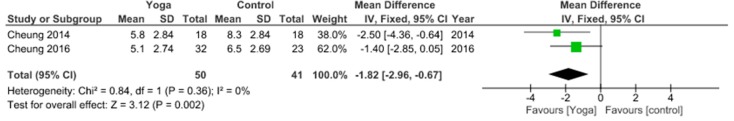
Pain: Yoga versus Control.

Almost all the included studies assessed pain through WOMAC pain and KOOS pain scale. The meta-analysis showed no significant pain decrease (SMD 0.33, 95% CI 0.03 to 0.63) and no heterogeneity (I^2^ = 0%) when comparing aquatic exercise and land-based exercise (Figure 2). Compared to controls for pain reduction, aquatic exercise presented significant differences (SMD −0.53, 95% CI −1.25 to 0.19) and high heterogeneity (I^2^ = 91%) (Figure 3), land-based exercise demonstrated no significant differences (SMD −0.26, 95% CI −0.42 to −0.11) and no heterogeneity (I^2^ = 0%) (Figure 4), Tai Chi reported significant differences (MD −2.14, 95% CI −3.11 to −1.18) and moderate heterogeneity (I^2^ = 38%) (Figure 5), Yoga had significant differences (MD −1.82, 95% CI −2.96 to −0.67) without heterogeneity (I^2^ = 0%) (Figure 6).

#### 3.7.2. Physical Function

**Figure 7:**
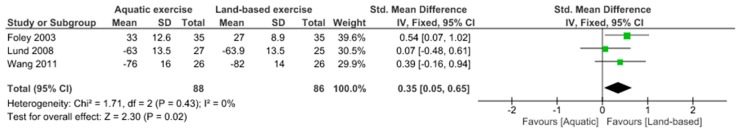
Function: Aquatic exercise versus Land-based exercise.

**Figure 8:**
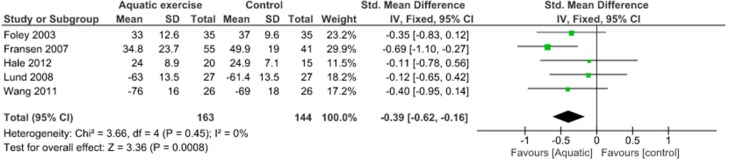
Function: Aquatic exercise versus Control.

**Figure 9:**
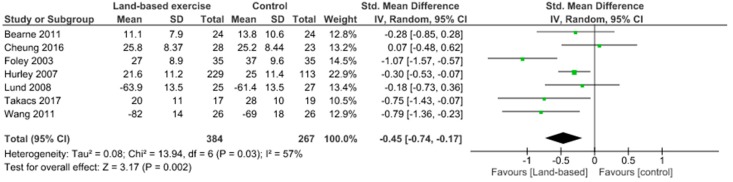
Function: Land-based exercise versus Control.

**Figure 10:**
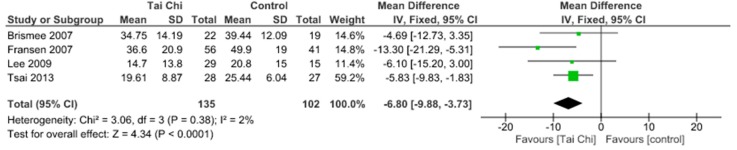
Function: Tai Chi versus Control.

**Figure 11:**
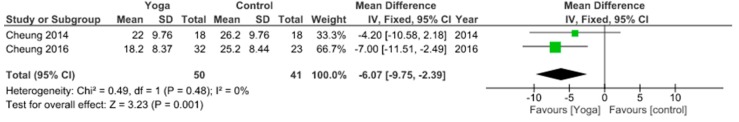
Function: Yoga versus Control.

Physical function was assessed using the WOMAC physical function scale and KOOS ADL. It has been demonstrated no significant physical function improvements (SMD 0.35, 95% CI 0.05 to 0.65) and no heterogeneity (I^2^ = 0%) between aquatic exercise and land-based exercise (Figure 7). Compared to controls for increase of physical function, aquatic exercise showed significant differences (SMD −0.39, 95% CI −0.62 to −0.16) and no heterogeneity (I^2^ = 0%) (Figure 8), land-based exercise presented significant differences (SMD −0.45, 95% CI −0.74 to −0.17) and high heterogeneity (I^2^ = 57%) (Figure 9), Tai Chi had significant differences (MD −6.80, 95% CI −9.88 to −3.73) and low heterogeneity (I^2^ = 2%) (Figure 10), Yoga reported significant differences (MD −6.07, 95% CI −9.75 to −2.39) without heterogeneity (I^2^ = 0%) (Figure 11).

#### 3.7.3. Quality of Life

**Figure 12:**
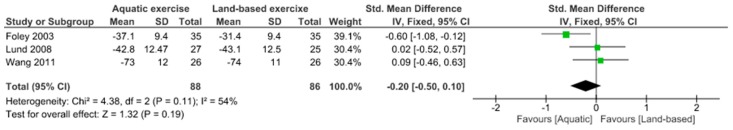
Quality of Life: Aquatic exercise versus Land-based exercise.

**Figure 13:**
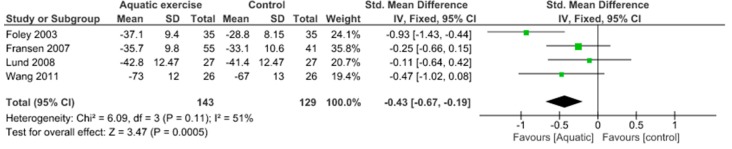
Quality of Life: Aquatic exercise versus Control.

**Figure 14:**
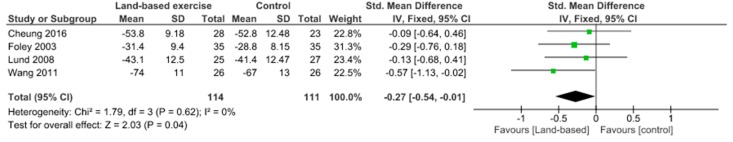
Quality of Life: Land-based exercise versus Control.

Quality of life was evaluated by KOOS QOL and SF-12. The meta-analysis showed no significant improvement in quality of life (SMD −0.20, 95% CI −0.50 to 0.10) and moderate heterogeneity (I^2^ = 54%) in the aquatic exercise compared to land-based exercise (Figure 12). Compared to controls for effect on the quality of life, aquatic exercise produced significant differences (SMD −0.43, 95% CI −0.67 to −0.19) and moderate heterogeneity (I^2^ = 51%) (Figure 13), while land-based exercise reported no significant differences (SMD −0.27, 95% CI −0.54 to −0.01) and no heterogeneity (I^2^ = 0%) (Figure 14).

#### 3.7.4. Stiffness

**Figure 15:**
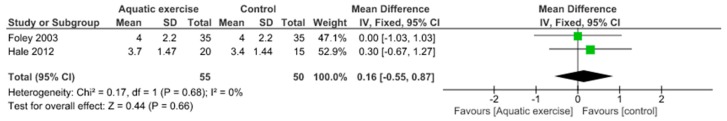
Stiffness: Aquatic exercise versus Control.

**Figure 16:**
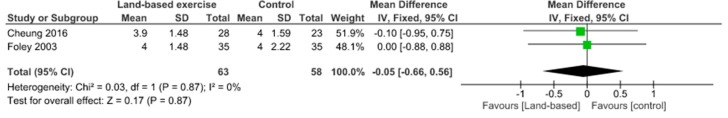
Stiffness: Land-based exercise versus Control.

**Figure 17:**
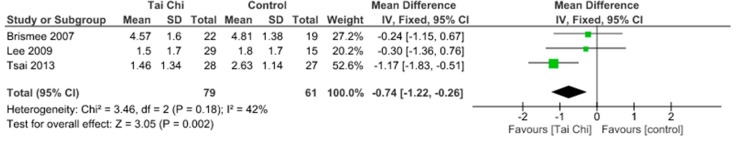
Stiffness: Tai Chi versus Control.

**Figure 18:**
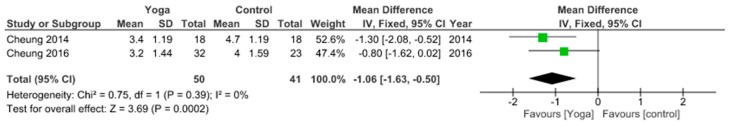
Stiffness: Yoga versus Control.

Stiffness was checked with the WOMAC stiffness scale. The meta-analysis presented no significant reduction of stiffness (MD 0.16, 95% CI −0.55 to 0.87) and no heterogeneity (I^2^ = 0%) in the aquatic exercise compared to the control group (Figure 15). Similarly, in the land-based exercise, there was no significant decrease of stiffness (MD −0.05, 95% CI −0.66 to 0.56) and no heterogeneity (I^2^ = 0%) compared to the controls (Figure 16). Compared to controls improvement of stiffness, Tai Chi showed significant differences (MD −0.74, 95% CI −1.22 to −0.26) and moderate heterogeneity (I^2^ = 42%) (Figure 17), and Yoga reported significant differences (MD −1.06, 95% CI −1.63 to −0.50) without heterogeneity (I^2^ = 0%) (Figure 18).

#### 3.7.5. Dynamic Balance

**Figure 19:**
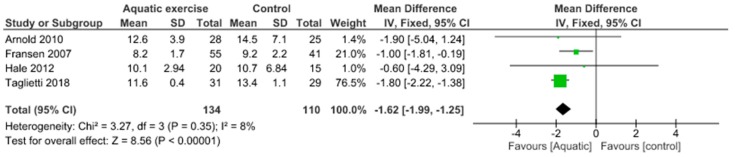
Dynamic Balance: Aquatic exercise versus Control.

Only aquatic exercise studies evaluated the dynamic balance by the Time Up and Go test. It has been reported significant improvement of dynamic balance (MD −1.62, 95% CI −1.99 to −1.25) and low heterogeneity (I^2^ = 8%) in the aquatic exercise in comparison with controls (Figure 19).

### 3.8. Quality Assessment

The quality of the evidence of the included studies was assessed for 18 comparisons using the GRADE system (Table 5). Of these,15 comparisons were downgraded by one level due to serious risk of bias, especially as regarded the lack of blinding; therefore, they presented a moderate quality. The remaining three comparisons were downgraded by two levels due to serious risk of bias and inconsistency because there was significant and unexplained variability in results from different trials.

## 4. Discussion

The primary aim of this systematic review and meta-analysis was to summarize the evidence of the efficiency of various types of physical activity on pain, physical function, stiffness, quality of life, and dynamic balance in patients aged 65 or over with knee and hip OA. Another endpoint was to examine either land-based active exercise, aquatic active exercise, and sports, in order to establish greater improvements. Physical activity has shown to be very beneficial for older people with knee and hip OA in terms of pain reduction, better function, performance, and quality of life, with statistically significant improvements compared to the control group. Nevertheless, it was not possible to determine with certainty greater long-term benefits of one type of physical activity compared to the others, also considering the different rates of adherence, and adverse events. The literature review produced almost exclusively RCTs, and this could be justified by the fact that the follow-up was short, and after the follow-up, the control group could have received the same training program as the treatment group. A limitation of this study was represented by the fact that only five studies were composed of three arms, of which two intervention groups and one control group. Three studies (32–34) with two intervention groups presented in the quantitative analysis quite contrasting results about variations in pain and physical function after treatments when comparing land-based with aquatic active exercise; however, showing improvements of both interventions compared to the control group. Also, when comparing Tai Chi or Yoga with aquatic and aerobic exercise [30,35], there were uncertain results on symptom improvement, pain relief, and perceived function. In five studies [44,45,47,48,49], active exercise represented the only intervention group, and in all of those participants in the study group showed significant improvement in the checked outcomes, such as WOMAC, KOOS, VAS, 6-MWT, and get-up-and-go test. On the other hand, only one study [43] reported no differences between the rehabilitation group and the control group in any outcome measure. When analyzing sports, such as Tai Chi, Yoga, or Baduanjin, all the studies presented significant improvements in pain, physical function, stiffness, and quality of life after the treatment. The quality of the RCTs was determined by the Cochrane Risk of Bias Tool. 18 out of 22 studies presented an unclear or high risk of bias, and it was caused by the inability of blinding personnel and participants when performing physical activity interventions, even more, if control groups underwent no intervention. In fact, the blinding tool of Cochrane Risk of Bias was inadequate or unclear in all the studies selected. Two case series [29,50] were evaluated using the MINORS score. They presented an average score of 11.5 points on a maximum of 24, influenced by the fact that they were non-comparative studies, thus resulting in 0 points in 4 of 12 items. Dong et al. [52] presented no significant difference for pain relief and physical function between aquatic exercise and land-based exercise for patients with knee OA, for both short- and long-term interventions. Goh et al. [53] studied the relative efficacy of different exercises for patients with knee and hip OA. In their systematic review, pain, function, and performance were significantly better with all types of exercise than usual care. Aerobic was the most beneficial exercise for pain and performance, whereas mind-body was also the best for pain and function. Moreover, strengthening and flexibility exercises improved multiple outcomes at a moderate level, while mixed exercise was the least effective for all outcomes, superior only to usual care. Bartels et al. [22] proved that aquatic exercise produced a small short-term advancement compared to no intervention in pain, disability, and quality of life for people with knee or hip OA. In a systematic review and meta-analysis by Lauche et al. [54], participants with knee osteoarthritis training Tai Chi presented an increase of pain, physical function and stiffness with moderate evidence and an increase of quality of life with strong evidence. Cheung et al. [51] demonstrated the effectiveness of yoga to reduce pain, stiffness, and swelling, even if, in their review, the results on physical function were inconclusive because of a variety of outcome measures being used. Another limitation of this review was due to the heterogeneity of the included studies, which presented different scores with various primary and secondary outcomes, different protocols—especially for aquatic and land-based exercise—with distinct types of exercise, different timings of follow-up with variable duration of the single session and the entire program. Therefore, it was not possible to clearly compare the obtained results of the different study groups, although it was evident the efficacy of every type of physical activity compared to the control groups. Furthermore, adherence to physical activity was reported by 14 of the included studies. In these studies, the adherence to the programs was high, with an attendance of about 80% at the rehabilitation sessions, without significant differences between land-based and aquatic exercise and sport, although that attendance was higher in hydrotherapy when compared with other intervention groups [30,37,38]. The adherence to a physical exercise regimen is essential in order to improve physical performance and function and reduce pain, especially in older patients. Van Gool et al. [55] proved that higher exercise adherence leads to improvements in physical performance and self-reported disability in older adults with knee OA. All the included studies presented the details of dropouts. Twelve studies reported the adverse events that happened over the treatment period. In 4 studies no side effects, complications, or injuries were reported during the physical program [33,34,41,45]. Muscle soreness, increased foot and knee pain, and low back pain after exercise were the most common adverse effect, while they were recorded in a few patients [31,35,36,39,42,49]. Arnold et al. [42] described one moderate adverse effect, that consisted of spinal pain due to a fall. In a study by Fransen et al. [30], 11 participants presented a serious adverse, which were not related to the intervention. Lund et al. reported [38] 11 adverse effects in the land-based group and three adverse effects in the aquatic group (*p* = 0.012).

## 5. Conclusions

This review and meta-analysis show that all active exercise and sport are an effective conservative treatment for elderly people with OA, in order to improve pain and physical function. The meta-analysis reported no significant differences in improvements in pain, physical function, and QOL between aquatic and land-based exercise. Compared to controls, the aquatic exercise showed significant differences for pain reduction and increase of physical function, quality of live and dynamic balance, land-based exercise presented significant differences for physical function, Tai Chi and Yoga demonstrated significant differences in improvements in pain, physical function, and stiffness. However, the number of studies in this research area is still too few to establish which physical activity leads to better improvement in pain, physical function, stiffness, and quality of life. More high-quality studies with a lower risk of bias are needed in order to support these results, and they should be designed as RCTs comparing aquatic exercise with land-based exercise and sport. Moreover, in future studies aquatic and land-based exercise should be standardized, with the creation of an exercise protocol explaining the program, the frequency and the duration of the exercise’s sessions and with the use of similar scores, in order to produce comparable data, to avoid dropouts and to increase the adherence to the programs.

## Figures and Tables

**Table 1:** Demographic data of the included studies.

Author (Year)	Type of Study	LOE	Study Group				Control Group				Joint/s
			*n*	Age	Sex	BMI	*n*	Age	Sex	BMI	
Arnold et al. (2010) [42]	RCT	I	Aquatic and education: 28	73.2 y	71.4% F, 28.6% M	29.2	25	75.8 y	64% F, 36% M	30	hip OA
			Aquatic: 26	74.4 y	77% F, 23% M	30.4					
Bearne et al. (2011) [43]	RCT	I	24	65 y	62.5% F, 37.5% M	27.3	24	67 y	79% F, 21% M	26.9	hip OA
Bezalel et al. (2010) [44]	RCT	I	25	73.8 y	68% F, 32% M	/	25	73.7 y	80% F, 20% M	/	knee OA
Brismee et al. (2007) [31]	RCT	I	22	70.8 y	86.4% F, 13.6% M	28	19	68.8 y	78.9% F, 21.1% M	27.7	knee OA
Casilda-López et al. ( 2017) [40]	RCT	I	17	65.62 y	100% F	31.69	17	66 y	100% F	33.65	knee OA
Cheung et al. (2014) [34]	RCT	I	18	71.9 y	100% F	29.1	18	71.9 y	100% F	28.8	knee OA
Cheung et al. (2016) [35]	RCT	I	Yog: 32	68.9 y	/	29.8	23	71.8 y	/	27.8	knee OA
			Exercises: 28	74.4 y	/	29.2					
Doi et al. (2008) [46]	RCT	I	63	67.4 y	76% F, 24% M	24.8	58	71.2 y	72% F, 28% M	24.3	knee OA
Foley et al. (2003) [37]	RCT	I	Aquatic: 35	73 y	43% F, 57% M	/	35	6.8 y	57% F, 43% M	/	hip and knee OA
			Land-based: 35	69.8 y	49% F, 51% M	/					
Fransen et al. (2007) [30]	RCT	I	Aquatic: 55	70 y	73% F, 27% M	30	41	69.6 y	83% F, 17% M	30.7	hip and knee OA
			Tai chi: 56	70.8 y	68% F, 32% M	29.6					
Hale et al. (2012) [39]	RCT	I	23	73.6 y	74% F, 26% M	/	16	75.7 y	75% F, 25% M	/	hip and knee OA
Huang et al. (2017) [45]	RCT	I	128	68.07 y	79% F, 21% M	24.11	122	67.42 y	80% F, 20% M	25.01	knee OA
Hurley et al. (2007) [48]	RCT	I	Individual rehabilitation: 146	66 y	71% F, 29% M	30	140	67 y	68.5% F, 31.5% M	30.3	knee OA
			Group rehabilitation: 132	68 y	71% F, 29% M	30.18					
Lee et al. (2009) [32]	RCT	I	29	70.2 y	93.1% F, 6.9% M	26	15	66.9 y	93.3% F, 6.7% M	26	knee OA
Lund et al. (2008) [38]	RCT	I	Aquatic: 27	65 y	83% F, 17% M	27.4	27	70 y	66% F, 34% M	26.1	knee OA
			Land-based: 25	68 y	88% F, 12% M	23.7					
Marconcin et al. (2017) [47]	RCT	I	35	70.3 y	80% F, 20% M	32.3	32	67.8 y	59.4% F, 40.6% M	30.1	knee OA
Taglietti et al. (2018) [41]	RCT	I	31	67.3 y	74.2% F, 25.8% M	29.2	29	68.7 y	62.1% F, 37.9% M	30.4	knee OA
Takacs et al. (2017) [49]	RCT	I	20	66,1 y	95% F, 5% M	28.5	20	67.1 y	65% F, 35% M	28.9	knee OA
Tsai et al. (2013) [33]	RCT	I	28	78.89 y	78.6% F, 21.4% M	/	27	78.93 y	66.7% F, 33.3% M	/	knee OA
Wang et al. (2011) [36]	RCT	I	Aquatic: 26	66.7 y	84.6% F, 15.4% M	/	26	67.9 y	84.6% F, 15.4% M	/	knee OA
			Land-based: 26	68.3 y	88.5% F, 11.5% M	/					
An et al. (2013) [50]	CS	IV	22	66 y	86% F, 14% M	25	/				knee OA
Bove et al. (2017) [29]	CS	IV	7	66 y	71.5% F, 28.5% M	30.5	/				knee OA

RCT: Randomized Clinical Trial; CS: Case Series; LOE: Levels of Evidence; BMI: Body Mass Index; OA: Osteoarthritis; *n*: Number of participants; y: years; F: female; M: male.

**Table 2:** Clinical outcome data of the included studies.

Author (Year)	Intervention(s)	Control	Primary Outcome Measure	Secondary Outcome Measure	Follow-Up	Results
Arnold et al. (2010) [42]	Aquatic and education: aquatic exercise twice a week with once-a-week group education for 11 weeks; Aquatic: two weeks aquatic exercise for 11 weeks	no intervention	Berg Balance Scale, 6-MWT, Timed Up and Go Test	PASE score, AIMS-2 score	11 weeks	Significant improvement in fall risk factors (*p* = 0.038) with the combination of aquatic exercise and education.
Bearne et al. (2011) [43]	Ten 75-min group exercise and self-management sessions (twice a week for five weeks)	no intervention	WOMAC physical function	WOMAC pain, WOMAC total score	Six weeks and six months	No between-group differences in any outcome measure.
Bezalel et al. (2010) [44]	Group education program once a week for four weeks, followed by a self-executed home-based exercise program	six 20-min sessions of short-wave diathermy	WOMAC total score	Sit to stand test, Timed up and go test	four and eight weeks	Significant improvement in the timed up and go test and WOMAC total (*p* < 0.01) in the exercise group.
Brismee et al. (2007) [31]	Six weeks of group Tai Chi sessions, 40 min/session, three times a week, followed by another six weeks of home-based Tai Chi training	three 40-min group sessions per week for six weeks	WOMAC pain, VAS	WOMAC stiffness and physical function	3, 6, 9, 12, 15, and 18 weeks	Less overall pain and better WOMAC physical function with Tai Chi (*p* = 0.0089 and 0.0157, respectively).
Casilda-López et al. ( 2017) [40]	Eight-week dance-based aquatic exercise program	global aquatic exercise program	WOMAC total score	6-MWT and VAS	Eight weeks and Three months	Postintervention differences in the WOMAC pain and aggregate (*p* = 0.002 and *p* = 0.048) in favor of the experimental group.
Cheung et al. (2014) [34]	Eight-week Hatha yoga intervention involving group and home-based exercise sessions	no intervention	WOMAC total score	SPPB, SF-12	Four weeks, eight weeks and 20 weeks	Improvement in WOMAC pain *p* = 0.01) and stiffness (*p* = 0.02) in the intervention group.
Cheung et al. (2016) [35]	Yoga: one 45-min class per week for eight weeks and additional 30 min/day, four times/week of yoga practice at home; Exercises: eight weekly group-based classes	no intervention	WOMAC total score, VAS	SPPB, SF-12	Four and eight weeks	Yoga group presented improvements in WOMAC TOTAL (*p* = 0.001) and VAS scores (*p* = 0.03) compared to exercises group.
Doi et al. (2008) [46]	Four sets of 20 repetitions of quadriceps exercise every day (knee extension movements while sitting on a chair or in a supine position)	NSAIDs	WOMAC total score and VAS	SF-36	Eight weeks	Improvements in total WOMAC, SF-36 and VAS: all *p* < 0.001 in the exercises group; WOMAC and VAS at *p* < 0.001 and SF-36 at *P* < 0.03 in the control group.
Foley et al. (2003) [37]	Three water based, or three gym-based exercise sessions a week for six weeks, including a short warm up period, lower limb stretches, and a set of resistance exercises	no intervention	WOMAC total score, 6-MWT	SF-12	Six weeks	Walking speed and distance increased in the hydrotherapy and gym groups (both *p* < 0.001). No significant changes for WOMAC function or stiffness.
Fransen et al. (2007) [30]	Aquatic or Tai Chi program (with a preliminary 10-min warm-up session): 1 h, twice a week for 12 weeks	no intervention	WOMAC pain and physical function	SF-12, DASS21	12 and 24 weeks	Improvements of 6.5 and 10.5 for pain and physical function scores with hydrotherapy and improvements of 5.2 and 9.7 with Tai Chi.
Hale et al. (2012) [44]	Water-based exercise classes twice weekly for 12 weeks	community-based computer-skills training program	PPA	Step Test, Timed Up and Go Test, WOMAC total score	12 weeks	No statistically significant between-group differences were found for any outcome measured.
Huang et al. (2017) [45]	Quadriceps isometric contraction exercise (two sets of exercises in the morning and evening)	local physiotherapy and oral NSAIDs	WOMAC total score, VAS	/	One and three months	Significant improvement of WOMAC and VAS score in the experimental group (*p* < 0.05).
Hurley et al. (2007) [48]	12 supervised sessions that combined discussion on specific topics regarding self-management and coping, with an individualized, progressive exercise regimen	no intervention	WOMAC physical function	WOMAC pain, WOMAC total score	Six weeks and six months	Individual and group rehabilitated participants had better WOMAC score (*p* = 0.01) than control group.
Lee et al. (2009) [32]	Eight weeks of group Tai Chi Qigong sessions, with 60 min per session twice a week	no intervention	SF-36	WOMAC total score, 6-MWT	Eight weeks	Improvements in SF-36 (*p* = 0.010) and 6- MWT (*p* = 0.005) in the Tai Chi group.
Lund et al. (2008) [38]	Aquatic and land-based exercise programs for eight weeks with 2 sessions per week	no intervention	KOOS function and pain, VAS	Muscle Strength	Eight weeks and three months	Only in the land-based exercise group a decrease of pain was detected (*p* = 0.039). There were no significant differences between groups for KOOS.
Marconcin et al. (2017) [47]	PLE2NO program: 90-min intervention twice a week for 12 weeks	no intervention	KOOS pain	KOOS function and total score, 6-MWT	12 weeks	Significant clinical improvement was found for all KOOS (larger than 10 points) and in the 6 MWT (*p* = 0.035) in the exercise group.
Taglietti et al. (2018) [41]	Aquatic program twice a week for eight weeks	educational program: once a week for eight weeks	WOMAC total score, VAS	SF-36, Timed up and go test, Yesavage Geriatric Depression Scale	Three months	WOMAC pain reduced in favour of the aquatic exercise group (*p* = 0.021). No differences for the outcome’s functional mobility or depression.
Takacs et al. (2017) [49]	Ten weeks of exercises targeting dynamic balance and strength performed four times per week	no intervention	CB&M, WOMAC physical function	Muscle Strength	10 weeks	Improvements in self-reported pain (*p* = 0.005), physical function (*p* = 0.002), and fear of movement (*p* = 0.01) in the training group.
Tsai et al. (2013) [33]	Three sessions a week of Tai Chi exercise (12-form Sun Tai Chi) for 20 weeks	no intervention	WOMAC pain	WOMAC physical function and stiffness, timed up and go test and Sit to stand test	21 weeks	WOMAC pain (*p* < 0.001), physical function (*p* = 0.001) and stiffness scores (*p* = 0.001) improved in the Tai Chi group.
Wang et al. (2011) [36]	Aquatic/land-based exercise protocol with a 60-min flexibility and aerobic training class, three times a week for 12 weeks	no intervention	KOOS total score, 6-MWT	knee ROMs	Six and 12 weeks	Aquatic and land group presented less pain than control group (respectively *p* < 0.001 and *p* = 0.002).
An et al. (2013) [50]	Short-term Baduanjin exercise: 30-min sessions five times a week for one year	/	WOMAC total score, SF-36	6-MWT	one year	WOMAC pain, stiffness and physical function subscales, SF-36 body pain and 6-MWT were significantly improved.
Bove et al. (2017) [29]	16 sessions of task-specific training at a frequency of two visits per week	/	KOOS total score	30-Second Chair Rise, Timed Stair Climb Test, Floor Transfer Test	Four, six, and eight weeks	Improvements in patient-rated and performance-based outcomes.

PASE: Physical Activity Scale for the Elderly; AIMS-2: Arthritis Impact Measurement Scales 2; WOMAC: Western Ontario and McMaster Universities Arthritis Index; VAS: Visual Analog Scale; 6-MWT: 6 min walk test; SPPB: Short Physical Performance Battery SF-12: Short Form-12; NSAIDs: Nonsteroidal anti-inflammatory drugs; DASS21: Depression, Anxiety and Stress Scale; PPA: Physiological Profile Assessment; KOOS: Knee injury and Osteoarthritis Outcome Score; CB&M: Community Balance and Mobility Scale; ROM: Range of Motion.

**Table 3:** Cochrane risk of bias tool for randomized controlled trials.

Study	Sequence Generation	Allocation Concealment	Blinding	Incomplete Data Addressed	Free of Selective Reporting	Free of Other Bias	Risk of Bias
Arnold et al. (2010)	L	L	H	L	U	L	B
Bearne et al. (2011)	L	U	H	L	U	U	C
Bezalel et al. (2010)	L	L	H	U	U	U	C
Brismee et al. (2007)	L	U	H	L	L	U	B
Casilda-López et al. ( 2017)	L	L	U	L	U	U	B
Cheung et al. (2014)	L	L	H	L	L	U	B
Cheung et al. (2016)	L	L	H	L	L	L	A
Doi e al. (2008)	L	L	H	H	L	U	B
Foley et al. (2003)	L	L	U	L	L	U	B
Fransen et al. (2007)	L	L	U	L	L	U	B
Hale et al. (2012)	L	L	U	U	L	L	B
Huang et al. (2017)	U	U	H	L	H	H	C
Hurley et al. (2007)	U	L	H	U	L	L	B
Lee et al. (2009)	L	L	H	L	L	L	A
Lund et al. (2008)	U	L	U	L	L	L	B
Marconcin et al. (2017)	H	U	H	L	L	U	C
Taglietti et al. (2018)	L	L	H	L	L	L	A
Takacs et al. (2017)	L	L	H	L	U	L	B
Tsai et al. (2013)	L	L	H	L	L	L	A
Wang et al. (2011)	L	U	H	L	L	L	B

**Table 4:** MINORS (Methodological Index for Non-Randomized Studies) score.

Study	Stated Aim	Inclusion of Patients	Collection of Data	Endpoints Appropriate to the Aim	Unbiased Assessment of the Study Endpoint	Follow-Up	Loss to Follow Up Less Than 5%	Prospective Calculation of the Study Size	Total
Am et al. (2013)	2	1	2	2	1	2	2	0	12
Bove et al. (2017)	2	1	2	2	1	1	2	0	11

**Table 5:** GRADE.

Outcomes	Comparisons	*n* of Participants (Studies)	Risk of Bias	Inconsistency	Indirectness	Imprecision	Other Considerations	Quality
Pain	Aquatic vs. Land-based	174 (3 RCT)	serious	not serious	not serious	not serious	not serious	⨁⨁⨁◯ moderate
Function	Aquatic vs. Land-based	174 (3 RCT)	serious	not serious	not serious	not serious	not serious	⨁⨁⨁◯ moderate
Quality of Life	Aquatic vs. Land-based	174 (3 RCT)	serious	serious	not serious	not serious	not serious	⨁⨁◯◯ low
Pain	Aquatic vs. Control	367 (6 RCT)	serious	serious	not serious	not serious	not serious	⨁⨁◯◯ low
Function	Aquatic vs. Control	307 (5 RCT)	serious	not serious	not serious	not serious	not serious	⨁⨁⨁◯ moderate
Quality of Life	Aquatic vs. Control	272 (4 RCT)	serious	not serious	not serious	not serious	not serious	⨁⨁⨁◯ moderate
Stiffness	Aquatic vs. Control	105 (2 RCT)	serious	serious	not serious	not serious	not serious	⨁⨁◯◯ low
Dynamic Balance	Aquatic vs. Control	244 (4 RCT)	serious	not serious	not serious	not serious	not serious	⨁⨁⨁◯ moderate
Pain	Land-based vs. Control	682(7 RCT)	serious	not serious	not serious	not serious	not serious	⨁⨁⨁◯ moderate
Function	Land-based vs. Control	651 (7 RCT)	serious	not serious	not serious	not serious	not serious	⨁⨁⨁◯ moderate
Quality of Life	Land-based vs. Control	225 (4 RCT)	serious	not serious	not serious	not serious	not serious	⨁⨁⨁◯ moderate
Stiffness	Land-based vs. Control	121 (2 RCT)	serious	not serious	not serious	not serious	not serious	⨁⨁⨁◯ moderate
Pain	Tai Chi vs. Control	237 (4 RCT)	serious	not serious	not serious	not serious	not serious	⨁⨁⨁◯ moderate
Function	Tai Chi vs. Control	237 (4 RCT)	serious	not serious	not serious	not serious	not serious	⨁⨁⨁◯ moderate
Stiffness	Tai Chi vs. Control	140 (3 RCT)	serious	not serious	not serious	not serious	not serious	⨁⨁⨁◯ moderate
Pain	Yoga vs. Control	91 (2 RCT)	serious	not serious	not serious	not serious	not serious	⨁⨁⨁◯ moderate
Function	Yoga vs. Control	91 (2 RCT)	serious	not serious	not serious	not serious	not serious	⨁⨁⨁◯ moderate
Stiffness	Yoga vs. Control	91 (2 RCT)	serious	not serious	not serious	not serious	not serious	⨁⨁⨁◯ moderate

RCT: Randomized Clinical Trial.

## Supplementary Materials

The following are available online at https://www.mdpi.com/2077-0383/9/4/1167/s1, Figure S1: PRISMA Checklist.
